# Multidisciplinary Approach and Outcomes of Tele-neurology: A Review

**DOI:** 10.7759/cureus.4410

**Published:** 2019-04-08

**Authors:** Urvish K Patel, Preeti Malik, Matthew DeMasi, Abhishek Lunagariya, Vishal B Jani

**Affiliations:** 1 Neurology, Icahn School of Medicine at Mount Sinai, New York, USA; 2 Pediatrics, The Children's Hospital at Montefiore, Bronx, USA; 3 Internal Medicine, Albert Einstein College of Medicine, Bronx, USA; 4 Neurology, Creighton University School of Medicine, Omaha, USA

**Keywords:** tele-neurology, stroke, epilepsy, neurology, outcomes, literature reviews, tele-medicine

## Abstract

In rural and underserved areas, there are restrictions in healthcare due to the lack of availability of neurologists; patients have to travel long distances to receive the required care. Considering the fact that neurological conditions have large mortality and disability rates, there is a need for innovative services like tele-neurology. It is an important tool in improving the health and quality of life by using different ways of communication between neurologists and patients, or neurologists and other providers. We examine the current types of facilities available in tele-neurology, as well as outcomes, barriers, limitations, legal litigations, and the multidisciplinary nature based on prior studies. We have also suggested recommendations for the future of tele-neurology including effective-accessibility and inexpensive-utilization in developing countries.

There are various tele-health programs created by The Veterans Health Administration including a clinical video tele-health (CVT) system. This system allows direct patient care of veterans by neurologists. The University of South Carolina implemented a web-based tele-stroke program in which acute ischemic stroke patients were treated in the Emergency Department (ED) of rural hospitals by neurologists, after consulting with rural ED physicians. With growing technology and popularity of tele-neurology, there are now international collaborative efforts in tele-medicine that are looking to be adapted to tele-neurology. Thus, tele-neurology can provide quality neurological care with patient satisfaction, as well as time and cost savings. The tele-stroke group established by TRUST-tPA trial (Therapeutic Trial Evaluating Efficacy of Telemedicine (TELESTROKE) of Patients With Acute Stroke) has 10 community hospital-emergency rooms that were connected to a stroke center. It was found that tele-stroke is appropriate in places where there is no way for a patient to access a stroke unit within a 4.5-hour time window. Like other tele-neurology subtypes, tele-epilepsy and pediatric tele-neurology also offer more follow-up care to people of remote areas which have limited access. There are other subtypes like mental health, chronic neurological care, and hospitalist which are very effective in improving outcome and quality of life of people living in remote areas. Tele-neurology has effectively reduced travel costs and times; there is high patient satisfaction and reduced disparity for general and specialized neurological care. But there are certain limitations like large equipment costs, certain bandwidth requirement, and trained staff to use the equipment. Transmission of patient information using public internet raises the concern of legality. There should be sufficient encryption to satisfy the Health Insurance Portability and Accountability Act (HIPAA) requirements to ensure patient confidentiality and safety of personal data.

The adaptation of tele-neurology is a powerful and innovative way to enhance healthcare in areas with a shortage of specialists. Implementation of this tool is limited due to cost burden, lack of expertise to implement necessary technology, legal litigations, and suitable financial and professional incentives for the users. This review focuses on the trajectory of utilization and the issues to be addressed in order to provide the full benefits of tele-neurology to undeserved communities in the future.

## Introduction and background

Tele-neurology is the use of real-time or delayed communication between neurologists and patients, or neurologists and other providers by way of shared audio, video, secure messaging, and other data exchanges which has been an important tool in improving the health and quality of life in those who are restricted to quality neurological care [1]. These restrictions to care are often due to geographic isolation; there are certain areas that lack a neurologist or patients must travel long distances to receive care [2-3]. There is a significant disparity between the need and availability of neurologists, especially in rural and underserved communities [4-5]. With neurological conditions continuing to be large contributors to mortality and disability, there needs to be new and innovative services, such as tele-neurology, to combat these problems and supply this need [1,5].

Tele-neurology has provided an outlet in which patients and physicians, although at great distances apart, are able to communicate and be examined within an instant. This is relevant for chronic conditions that require continuous follow-up, or in emergencies, in which the administration of thrombolytic therapy in stroke, for example, is often needed within a short time window to be effective [2]. Tele-neurology has the potential to reduce healthcare costs, hospitalization services, and improve multidisciplinary communication between different providers regarding the care for their patients.

In this review, we have examined the current types of tele-neurology available, as well as prior research, outcomes, limitations, and the multidisciplinary nature of the relevant subtypes of tele-neurology including tele-stroke, tele-epilepsy, tele-radiology, pediatric tele-neurology, general tele-neurology, mental health, chronic neurological care, and tele-neurohospitalist work. We have also suggested recommendations for the future of tele-neurology including effective-accessibility and inexpensive-utilization in developing countries.

## Review

Types of tele-neurology facilities/consultations

Tele-neurology can take two forms: synchronous or asynchronous. Synchronous forms of tele-neurology are those in which patients and clinicians connect in real-time. Asynchronous tele-neurology, which is also called “store and forward,” is one in which there is delayed communication. Clinical information is collected, and the data is then transmitted electronically and reviewed later by the clinician. This can include the transmission of digital photos, videos, or data files for review or evaluation [6-8].

The Veterans Health Administration created a variety of tele-health programs, including a clinical video tele-health (CVT) system which enabled neurologists to directly talk to and examine veterans with neurological illnesses. The patient would arrive at a local community-based outpatient clinic and be examined remotely by a neurologist using a real-time tele-health camera. Each visit included time for medical history and an examination such as mental status exam, cranial nerve exam, and motor evaluation. Test results, disease education, and treatment plans would also be discussed during the visit [9]. Similarly, the University of Kansas Center for Tele-medicine and Tele-health provides a pediatric neurology tele-medicine clinic that uses real-time videoconferencing equipment so that a pediatric neurologist can examine and observe nonverbal behavior in real-time with patients at a distant clinic. There is an on-site coordinator and distant site coordinator to assist the patient and family, to ensure that the technology is functioning properly, and to attain vital signs. There are 80 sites across Kansas, and most people are able to identify a site within an hour from their home [10].

Thus, tele-neurology can exist as a local hub or facility that is connected electronically to a larger hospital, neurologist, or specialist to provide consultation. The audio-visual conferencing equipment often consists of a camera with pan, tilt, and zoom capabilities so that the neurologist or specialist can examine and speak to the patient [11]. Other sorts of devices used to evaluate patients remotely include using robotic telepresence (RTP) in which a robotic audiovisual platform can move around to create a sense of “being present” at the remote site [12]. However, it does not need to be an outpatient facility which houses the tele-neurology consultation; it can also take place in rural hospitals. For instance, The University of South Carolina was able to implement a web-based tele-stroke program in which neurologists were able to consult rural ED physicians and nurses on patients presenting with acute ischemic stroke at these rural hospitals [13]. This system enabled the neurologist to have both an interaction with the patient and family, as well as a consultation with other providers.

This sort of interactive videoconferencing in which patients, relatives, and providers can all interact at the bedside and remotely communicate in real time is a huge shift from the more historical methods of tele-neurology. In fact, telephone consultation can be considered one of the simplest forms of tele-medicine in which a physician consults other providers on patient symptoms and management [14]. There have even been studies in which telephone consultation was used and found to be acceptable in identifying critical clinical neurological events that would necessitate face-to-face consultation in children with neuro-cysticercosis [15]. Thus, although the trend seems to be shifting toward interactive audio-videoconferencing, tele-neurology can exist in many other forms.

For instance, consultations can be carried out through email; an email tele-neurology service for patients referred to a neurologist by general practitioners was initiated in Northern Ireland. The neurologist would receive an email referral, and then decide whether advice alone was appropriate, or whether further investigations or a clinic visit was warranted. This was proven to improve clinical effectiveness, lower direct costs, and increase productivity [16].

Tele-neurology can also take the form of a web-based messaging system. For instance, Médecins Sans Frontières (MSF), which is a medical humanitarian emergency nonprofit organization, provides doctors at its field sites with access to a wide range of specialist consultations, including neurologists, through a web-based messaging system; only internet access and a computer is required [6]. Expanding on this web-based messaging system, WhatsApp has also been used as a clinical communication tool; WhatsApp is an instant messaging app available on smartphones that allows free text messages, voice calls, videoconferences, and file exchanges. In developing and economically limited countries, audio-visual conferencing systems and proper communication platforms are often not feasible. WhatsApp is used for organization, sharing clinical data, and clinical care guidance. For instance, in stroke care, it allows the multidisciplinary care teams to be immediately notified of the arrival, location, and stage of evaluation for stroke patients [17].

Tele-neurology is also in use by way of new mobile technologies that provide real-time data tracking and assessments that create a sort of digital phenotype, in which biometric data can be monitored at the patient’s home and electronic health records can be incorporated into the examination [7,14,18]. Research is also being developed that may enable the use of smartphone technology to improve on measurements such as tapping speed and tremor severity in Parkinson’s disease, sensors to measure electroencephalogram (EEG) in those with epilepsy, and smartwatches that have the potential to detect and quantify seizures. The growing technologies will continue to expand into tele-neurology in an individualized and focused manner [7].

With growing technology, there are also now international collaborative efforts in tele-medicine that are looking to be adapted to tele-neurology. For instance, the Réseau en Afrique Francophone pour la Télémédecine (RAFT) network is a group of 10 French-speaking countries in Africa, along with France and Switzerland, that provides continuing medical education to healthcare professionals and tele-consultations to improve the quality and efficiency of the Sub-Saharan African health systems. Additionally, there is thet tele-health in Africa project which networks between European reference hospitals and African hospitals with the aim of improving medical care for those in low and middle-income countries [19]. Tele-neurology can eventually expand and spread so that these low-income countries can gain valuable consultations and healthcare knowledge for its growing neurological conditions.

Prior research

Tele-neurology has been shown to provide great benefit to populations with limited access to general and specialized neurological care, such as rural populations, and those with limited mobility, or deployed military members [4].

Around 41% of all veterans enrolled in the Veterans Affairs healthcare system live in rural areas, and 27% of these have a disability or chronic medical condition; many of the healthcare needs of these veterans are also not being adequately met. As a result, the Veterans Health Administration created a large national tele-health network and was able to provide over 1 million episodes of care in 2012. Community-based outpatient clinics were developed in rural towns throughout the United States and this tele-medicine system was adopted in which patients could be remotely examined by a specialist, including a neurologist. Veterans with chronic neurological conditions were offered follow-up tele-neurology care at the clinics; it was found that patients were highly satisfied with the convenience and quality of their visit and that neurology providers believed tele-neurology was equivalent to clinic follow-up. An average time savings of five hours and 325 miles driven were calculated, and a total of $48,000 was saved in total costs; thus, tele-neurology to rural veterans can provide quality neurological care, with patient satisfaction, as well as time and cost savings [9,20].

In addition to providing rural care to chronic neurological conditions, tele-neurology has also been proven to be effective in providing stroke care to patients. Acute ischemic stroke patients can be administered alteplase (tPA) within 4.5 hours of symptom onset in order to reduce long-term disability; reduced times to administration of tPA are associated with better long-term functional outcomes and lower rates of complications [21]. Unfortunately, door to needle times have also been shown to be longer for rural patients due to their delay in presentation to a stroke center, and the inability of healthcare providers at rural hospitals to recognize stroke symptoms [21]. The TRUST-tPA trial (Therapeutic Trial Evaluating Efficacy of Telemedicine (TELESTROKE) of Patients With Acute Stroke) established a tele-stroke network of 10 community hospital emergency rooms that were connected to a stroke center; eligible patients were assigned to a usual care arm in which they were transferred to the stroke center for intravenous recombinant tissue plasminogen activator (IV rt-PA) administration, or to the tele-thrombolysis arm in which patients were evaluated by tele-neurology, administered IV rt-PA, and then transferred to a stroke center. It was found that tele-stroke is appropriate in places where there is no way for a patient to access a stroke unit within a 4.5 hour time window [22].

Beyond stroke, Rubin et al. conducted a systematic review and found that the use of tele-medicine for general and subspecialty neurological consultation appears very promising [23]. For instance, Müller et al. found that tele-medicine patients did not express less long-term satisfaction one year after specialist evaluation for non-acute headache. It has also been shown, through a Northern Ireland group, that tele-neurology is suitable for neurological outpatient referral, and that live video-link telemedicine consultation is associated with shorter hospital stays for patients with neurological disorders admitted under the care of non-neurologists in remote hospitals [24-25].

This group conducted its work in Northern Ireland, where there is a shortage of neurologists and a dispersed rural area so that many people with neurological illnesses are seen by general physicians, rather than neurologists. This, like the Veterans Health Administration providing tele-neurology services to rural veterans, enables a fruitful environment for the implementation of telemedicine. However, it has also been shown that tele-neurology can be useful in urban areas as well, and have resulted in a more organized neurologist workflow, accurate prehospital assessment, and psychosocial support [1]. A general tele-neurology program was established in Long Beach, California in which encounters were performed through clinical video tele-health with patients located at one of four community-based outpatient clinics located at short distances from the main facility. Despite the short distance, patients were satisfied with their care and preferred clinical video tele-health over face-to-face encounters; this, along with patient’s consistently keeping appointments and rare technical problems, demonstrated the successful application of tele-neurology in the management of diverse neurological disorders in an urban setting [26].

Despite the barriers some patients face in accessing traditional face-to-face neurology care such as traveling large distances or having a disability, in a survey of local physicians of Parkinson’s patients, many were ambivalent about recommending tele-health visits for their patients. Many also reported not receiving recommendations of care from the tele-neurologist, but of those who did, they implemented some or all of their recommendations and found them to be beneficial [11]. Despite this, tele-neurology is still growing; in a survey to neurology departments around the United States, tele-neurology is being applied, by way of consultations, for acute stroke, movement disorders, neurocritical care, and chronic care [27].

In addition to this, tele-medicine has been shown to be a suitable tool for caring for the elderly by improving emotional status, decreasing depressive symptoms, and improving social functioning and cognitive levels [18]. Likewise, with the expansion of telecommunication infrastructure, internet service, and mobile phone penetration, tele-medicine has the potential to be effectively utilized in low and middle-income countries, and in bridging the rural-urban gap in access to specialized neurology services [19]. Even though these countries may be unlikely to have expensive videoconferencing equipment or integrated services digital network (ISDN) lines that are often necessary for neurologic exams or diagnoses in real-time video, inexpensive “store and forward” methods have been successfully implemented. For example, such a system was started between neurologists in the UK and a rehabilitation hospital in Bangladesh; a small team of staff were taught how to send email referrals to UK specialists who used their home-based email system. This successful program showed that “store and forward” tele-neurology is a feasible and practical option for providing neurological advice to clinicians in developing countries [28]. Various types of tele-neurology setups and their utilization have mentioned in Table 1.

**Table 1:** Types of tele-neurology setups and utilization details IV-tPA: intravenous tissue plasminogen activator; ED: emergency department; RAFT: Réseau en Afrique Francophone pour la Télémédecine; EEG: electroencephalogram; AIS: acute ischemic stroke.

Tele-neurology facilities/consultations:		
Type of model	Description	Usefulness
"WhatsApp" Mobile application [17]	In the developing and economically limited countries, the WhatsApp has been a solution and is used for organizations in the sharing of clinical data and clinical care guidance.	Allows the multidisciplinary care teams to be immediately notified of the arrival, location, and stage of evaluation for stroke patients. The communication on this application can be secured with end-to-end encryption.
Video Conference [9]	Neurologists providing direct and remote care to veterans with neurological illnesses.	Two-way, interactive, real-time video sessions at a bandwidth sufficient to allow for synchronous patient care.
Pediatric Video-conferencing [10]	There is an on-site coordinator and distant site coordinator to assist the patient and family to ensure that the technology is functioning properly, and to attain vital signs.	Pediatric neurologist can examine and observe nonverbal behavior in real time with patients at a distant clinic.
Audio visual conference (Tele-stroke) [13-14,22,29]	Neurologists and Radiologist are able to consult emergency physicians (ED) and nurses on patients presenting with acute ischemic stroke (AIS) at the rural hospitals to fasten the delivery of the treatment. After patient evaluation through video conferencing and review of imaging, recommendations regarding IV-tPA were communicated to the ED and followed American Heart Association/American Stroke Association guidelines.	This model enabled the neurologist to have both an interaction with the patient and family, as well as a consultation with other providers. It also allows for rapid evaluation of stroke patients and improved thrombolysis utilization rate and so the outcome of AIS patients.
Email consultations [16]	The neurologist would receive an email referral, and then decide whether advice alone was appropriate, or whether further investigations or a clinic visit was warranted.	This was proven to improve clinical effectiveness, lower direct costs, and increase productivity
International collaboration [19]	The RAFT network is a group of 10 French speaking countries in Africa, along with France and Switzerland, that provides continuing medical education to healthcare professionals and tele-consultations.	This improved the quality and efficiency of the Sub-Saharan African health systems and other middle- and low-income countries.
Smart phone and Smart watches [7]	Measurements such as mapping speed and tremor severity in Parkinson’s disease, sensors to measure EEG in those with epilepsy, and smartwatches that have the potential to detect and quantify seizures.	It provides a real time data tracking and assessments, in which biometric data can be monitored at the patient’s home and electronic health records, can be incorporated into the examination.
Clinical Video Telehealth (CVT) [26]	The tele-neurologists assess mental status, cranial nerves (motor functions), abnormal movements, coordination, and gait. The telehealth clinical technician (TCT) assists with evaluation of sensory function, muscle strength, tone, and tendon reflexes.	This model was successful in the management of diverse neurological disorders in an urban setting, as it showed patients satisfaction, preference over face-to-face encounters, keeping up with follow up appointments and rare technical problems.
Store and forward [28]	A small team of dedicated local staff in Bangladesh rehabilitation hospital was trained in the use of equipment and how to send email referrals to a series of UK specialists. The use of a numbering system ensured patient confidentiality.	With the help of this model, a great power of email as a simple, reliable, cheap and effective method of asynchronous communication was demonstrated. It also proved to be a feasible and practical option for providing neurological advice to clinicians in developing countries.

Tele-stroke

Stroke is a national health problem that is the third leading cause of death and leading cause of disability in the United States; 750,000 Americans experience a new or recurrent stroke each year and $30 million is spent annually in the United States on stroke-related medical expenses or lost wages [22]. The goal in acute ischemic stroke is to preserve tissue, and the current strategy is the administration of alteplase as soon as possible [13,29]. However, fewer than 5% of acute ischemic stroke patients receive intravenous thrombolytic drugs [19]. The reason behind this is that many physicians lack experience in administrating alteplase which has a 6% symptomatic hemorrhage rate, and because many patients live in rural or underserved areas where stroke unit care or an available neurologist is not available within the treatment window [2,13,29].

Tele-stroke has been indicated as a possible solution to these issues. Care is delivered by patients being sent to their local satellite hospitals where a neurologist can examine them through audio-visual conferencing and determine whether to administer thrombolytic therapy or if transfer to a stroke center is required. This has allowed more hospitals to manage acute strokes, and has increased the access to care for millions [7]. Systematic review of tele-stroke programs have found reduced mortality, beneficial health outcomes, and treatment reliability through video consultations [1]. It has been shown The National Institutes of Health (NIH) Stroke Scale can be reliably performed, that neurologists can reliably interpret unenhanced brain computed tomography (CT) images for stroke diagnosis, and that tPA can be safely administered through tele-neurology [4]. Thus, tele-stroke has enabled expertise care in rural settings, has increased access to thrombolysis for stroke patients, and there is evidence that it has improved long-term functional outcomes [14].

Tele-epilepsy

Epilepsy is the most common neurological condition that requires long-term follow-up; it affects 1% of the population including people of all ages, races, and economic backgrounds [10,24]. For people living in rural areas or those of lower socioeconomic status, adequate resources for diagnosis or care is often difficult since neurologists tend to practice in metropolitan communities [10].

A potential solution is providing tele-neurology services to these rural areas; since much of the diagnosis and monitoring of therapy in epilepsy is dependent on the patient’s history, tele-medicine can make a strong impact regarding diagnosis, management, and follow-up [15]. For instance, the first full-time epilepsy tele-medicine clinic was established in Western Canada and provides evidence for a high patient and carer satisfaction. Costs were also very low for the patient, and the tele-medicine clinic showed similar rates of seizures, hospitalizations, and emergency room (ER) visits as compared to conventional epilepsy clinics [14]. Other models in use include nurse-specialist run services; the nurse is supported through telephone tele-medicine with a neurologist and by real-time video-link if necessary, which has saved neurologist time and improved patient satisfaction [24]. Mobile technologies and remote monitoring are also taking place within epilepsy care; smartphone apps with the potential to detect seizures, quantify them, and notify care teams are emerging so to help combat unexpected death in these patients [7].

Thus, tele-epilepsy has the potential to offer more follow-up care to people of remote areas, as well as continuous monitoring that would not be possible before the emergence of technology and telemedicine. This will hopefully improve outcomes and better the life quality and lifespan of those with epilepsy.

Tele-radiology

Radiology and imaging studies are essential to neurology, as images can locate specific brain infarcts or tumors that can guide management. Thus, in order to be fully effective, tele-neurology needs to incorporate tele-radiology into its use. In fact, tele-radiology is an example which demonstrates how multidisciplinary tele-neurology truly has become and is becoming. For instance, tele-stroke programs in rural settings are refining their methods in order to best treat patients with thrombolytic therapy; one way to do this is by facilitating quick access to brain imaging when presenting with a stroke. These images can then be sent to the tele-neurologist or tele-radiologist and facilitate a diagnosis and management plan, which can include remote administration of thrombolytic therapy, remote rehab assessments, or further investigations like carotid ultrasound [14]. Many tele-stroke programs also now seek to reduce their door-to-CT times since these images are so vital in determining the patient’s stroke status [30]. Additionally, there are now mobile CT-enabled ambulances that can bring hospital-level care to the patient; thus, patients in rural areas or in crowded and congested cities can have imaging done in the ambulance and possibly have thrombolytic therapy administered within the time window since the delay in obtaining images will be reduced [7]. And with the emergence of technology and smartphones, tele-radiology imaging can be transmitted to handheld devices so as to improve rapid access to patient imaging [4]; thus, tele-neurologists, radiologists, local providers, and others can have increased access to patient imaging and results, and more quickly develop a multidisciplinary plan together.

Pediatric neurology

There are many pediatric neurological conditions, many of which require an early diagnosis for the treatment or prevention of associated cognitive or behavioral issues. Unfortunately, many pediatric neurologists tend to practice in metropolitan areas which present challenges for those in rural areas or those with limited transportation [10]. There are also exorbitant costs and often long wait times for neurologist care; for instance, the highest costs of childhood epilepsy are due to hospital services in the first year after diagnosis. However, costs can be reduced if those with uncomplicated epilepsy are followed by general physicians or pediatricians [15].

Due to the limited knowledge regarding protocols for epilepsy amongst general providers, a solution has been proposed using tele-medicine. After an initial evaluation, uncomplicated cases should be followed-up by telephone encounters with specialists; this would reduce costs, waiting periods, and specialist workload [15]. Likewise, in children and adolescents with mild-to-severe brain injury, tele-therapy though clinician-assisted computerized problem-solving training was found to lower levels of aggression, attention deficit hyperactivity disorder symptoms, and conduct disorder in school. This is important since children with brain injury, such as minor concussion, have been found to have long-term academic and functional challenges than those without injury [1].

Thus, like adult tele-neurology, pediatric tele-neurology has the potential to provide more care to those who would otherwise have difficulty accessing it. However, pediatric tele-neurology also offers the ability to be constructive in preventing further issues by providing care faster and more continuously in hopes to improve pediatric neurological and behavioral symptoms, which could impact further care later in life.

General neurology

In the developing and industrialized world, there is a shortage of neurologists to enable every patient with a neurological symptom to be seen by a neurologist if admitted to the hospital despite their being more accurate diagnoses and less poor outcomes associated with seeing a neurologist [24]. A solution to this problem is tele-neurology in which real-time videoconferencing can increase the number of patients seen by a neurologist, allow local providers to maintain realistic on-call schedules, or supply coverage when local offices are too busy [5]. In past studies, tele-neurology through videoconferencing has been shown to feasible for new and follow-up evaluations for a variety of neurological disorders and performed as well as face-to-face assessment for the diagnosis and planning of treatment [14,26]. In a comparison of standard telephone advice and audio-visual conferencing by a neurologist, audio-visual conferencing was found to increase quality and safety and led to a change of initial diagnosis in more than half of cases suggesting higher rates of diagnostic accuracy [31]. Tele-neurology has been proven to be feasible in the evaluation of movement disorders, multiple sclerosis, headache, dementia, and remote sleep assessments [5,7].

Tele-neurology can also be utilized in acute situations, such as in intensive care units (ICUs); through remote monitoring, ICU’s can expand coverage for emergency situations, as well as access subspecialty expertise in the management of neurological emergencies with studies finding reduced patient mortality and shortened lengths of stay [1,5]. Continuous video electroencephalography (cvEEG) monitoring may support critical care as well by providing teams with symptoms of seizure activity.

General tele-neurology has the potential to be effective in providing multidisciplinary care as many general neurological patients need these multi-faceted treatment modalities and care as opposed to the more traditional acute care tele-stroke model. To this point, there are tele-health peripheral devices that can monitor outcomes such as extremity range of motion assessments or wireless-enabled medication bottles to report medication adherence that are being adapted. Additionally, many mobile phone or tablet applications include symptom tracking, patient education materials, scheduling applications, online support groups, or health coaching. For example, many amyotrophic lateral sclerosis (ALS) patients have access to both tele-health videoconferencing services, as well as remote monitoring of pulmonary hygiene and web sites which allow the direct scheduling of tele-health services, chat room participation, and patient education [8]. Thus, tele-health is evolving from direct patient interaction or exams to one in which there is continuous monitoring of the patient’s health and interactive means to control their health.

Mental health

Over half of brain injury sufferers report problems such as depression, mood changes, anxiety, and have partaken in substance abuse as a coping mechanism [1]. Telemedicine has emerged as an effective assessment tool for sufferers of depression, substance abuse, and cognitive impairment, as well as can be impactful in combating anxiety and depression as people can gain quick access to a professional with just internet access [1,10].

Similarly, the elderly often suffer from loneliness and isolation as they lose functional autonomy, as well as cognitive, motor, and speech functions, and nutrition and personal hygiene. Senile depression is very common amongst the elderly, and they often eat excessively which can trigger chronic diseases such as cardiovascular disease and diabetes. Telemedicine can be a viable approach for this multi-disciplinary care by promoting remission of their depressive symptoms, improving social functioning, cognitive levels, and nutritional habits. Telemedicine can allow for physiological parameters to be continuously measured at home and can improve their moods and feelings of abandonment by having regular communication with others [18].

Mental health can be targeted in telemedicine by a neuropsychologist providing psychological support for the patient, by focusing on narrative and introspection training, or by empathy and identification of emotions. They can also strengthen cognition by focusing on orientation, relationships, and associations; attention training and memory enhancement by recognition tasks can also be targeted in these care models [18]. Mental health is thus an important component that can be addressed in the multidisciplinary model that is tele-neurology, and an important aspect which can reduce symptoms and lead to greater adherence to treatment plans, as well as cost reduction and satisfaction from patients and providers.

Chronic neurological care

Symptoms can be long-lasting and complex following brain injury, and cognitive symptoms such as attention disruption, memory impairment, and difficulty in executive functioning can lead to difficulties in social interaction, career performance, everyday activities, and accessing care [1,7]. Although these individuals with chronic conditions can benefit from neurologist care, the supply of neurologists is limited, and access to care is restricted by distance and patient disability [7]. Tele-heath services may ease the burden of traveling long distances and dealing with this myriad of symptoms; the services can be multidisciplinary and include video visits to medication management. They have been found to be effective and generate comparable outcomes to usual care.

For instance, Parkinson’s disease is a chronic condition that benefits from specialist care although 40% of Medicare beneficiaries with Parkinson’s do not see a neurologist [11]; a study showed that telemedicine could be effective in the chronic phase of neurological illness as patients with Parkinson’s showed improved outcomes in quality of life and motor performance via telemedicine. Similarly, virtual rehab devices with two-way communication to remotely sense paretic limb activity and provide active input to the limb are available [14].

Telemedicine can make a huge impact in these patients as fatigue levels can often impact the motivation to continue care; with this convenience of care, patient satisfaction will increase the motivation to continue therapy and lead to further compliance and improvement of symptoms.

Tele-neurohospitalist

Similar to outpatient problems, inpatient neurology has seen an upstroke in patients needing neurological care or consultation without the resources to provide this need. Neurohospitalists are site-specific neurological specialists dedicated to the hospital environment and consult and evaluate patients in the emergency room, critical care units, or on medical-surgical departments. However, there are not enough neurologists becoming neurohospitalists to meet demand, and tele-neurohospitalists can provide a solution [32].

Tele-neurohospitalists provide tele-neurology services by audio-visual conferencing to an emergency room or hospital remotely; they can be involved in general neurological consultations within the hospital or be involved in tele-stroke situations in deciding to administer thrombolytic therapy [32]. More studies are needed to test the reliability, safety, efficacy, and cost-effectiveness, but it expected to provide a similar quality of care and patient outcomes as neurohospitalists [33]. Thus, tele-neurohospitalists can add another dimension to quality patient care as all those with primary or associated neurological conditions can be evaluated, which can hopefully improve outcomes and reduce further visits.

Outcomes and limitations

As detailed above, there are numerous times in which tele-neurology has a positive impact. Exorbitant costs, for example, can be reduced from $140-180 for in-person acute care to about $40-50 for a tele-health session; neurology practices have found evidence of lowered patient out-of-pocket expenses and institutional costs savings with tele-health utilization [1]. There are also reduced travel costs and travel times associated with tele-neurology, as well as high patient satisfaction, reduced geographical disparity for general and specialized neurological care, and more educational opportunities for physicians available [4,26].

For all the benefits of tele-neurology, there are some issues and limitations as well. There are large upfront costs for equipment and telecommunication lines required for synchronous tele-neurology; the equipment must be operated by trained staff, and sufficient connection bandwidth is required and even then, some technology only allows one person to speak at each time. Additionally, many times the doctor does not have access to the electronic health record of the patient they are consulting [24-25]. Some imaging and tests are also not available remotely, and the ability to conduct the neurological exam is often limited. Thus, many tele-neurology practices only see a follow-up, rather than new, patients [9].

In regards to the neurological exam, the NIHSS stroke scale can be reliably performed through tele-neurology. Similarly, plantar responses, facial strength, sitting balance, sensation, and gait assessment through telemedicine are all strongly comparable to face-to-face examination. Likewise, post-stroke aphasia assessment and remote examination of Parkinson’s patient’s postural stability, speech, facial expression, and tremor at rest were all comparable. More moderate and fair interrater agreements by telemedicine, as opposed to face-to-face examination, include assessments of eye movements, deep tendon reflexes, power and coordination, finger taps, action tremor, and rigidity [14].

Asynchronous technology, although inexpensive and convenient for high-volume and triage work, does not allow for the neurologist to personally take a history or see the exam conducted [24]. Additionally, both synchronous and asynchronous tele-neurology have been viewed as promoting a shift within neurology from securing a final diagnosis to more a disease categorization type of work based on not-to-miss diagnoses. Clinicians have felt it is like “shooting in the dark at a moving target [6].”

There are also legality concerns associated with tele-neurology. Since transmissions traverse public Internet, there should be sufficient encryption to satisfy HIPAA requirements and to ensure patient confidentiality and the safety of personal data [5,14]. WhatsApp’s encryption, for instance, is not compliant and not considered safe even though this inexpensive and widespread tool is often used to communicate patient data; data is vulnerable once the phone is unlocked and data is not backed up by WhatsApp [17]. There is also the problem of writing prescriptions; in the United States, one needs a license to practice in the state where the patient is located. Thus, out of state providers are often unable to prescribe medication or order tests by tele-medicine [11,14].

Reimbursement is also frequently cited as a great challenge to tele-medicine; Medicare reimbursement is often difficult and requires certain service locations and appropriate sites to provide the healthcare, and many insurers only reimburse for in-person services [5,27]. No dominant reimbursement model has yet evolved for tele-medicine [27].

Perhaps the most striking limitation, however, is the view amongst some clinicians that tele-medicine leads to the loss of a personal touch, and a disruption of the doctor-patient relationship [18,27]. Benefits and barriers of tele-neurology have mentioned in Table 2.

**Table 2:** Benefits and barriers to tele-medicine implementations EEG: electroencephalogram; EMG: electromyography.

Telemedicine implementations
Benefits
Increased practice outreach, development, and efficiency
Decreased travel time and expenses for doctors and patients
Expansion of educational opportunities and continuing medical education for physicians
Individual and group education for patients about their neurologic disease
Easy recruitment of patients into clinical trials
Improvement of access to neurologic expertise for remote or underserviced areas
Reduction in geographical disparity for neurologic care
Decreased response time in stroke
High patient and family satisfaction survey scores with their tele-neurology care
Barriers
Disruption of traditional doctor–patient relationship
Physician reluctance to adopt novel technology in practice
Limitations to billing and reimbursement for time spent
Additional costs for technology
Licensing, credentialing issues for out-of-state physicians
Concern for malpractice liability
Performing complete neurologic examination solely via telehealth, particularly evaluating
Obtaining neurodiagnostic tests such as EEG, EMG, and neuroimaging in remote settings

Suggestions for tele-neurology implementations

Tele-medicine has some limitations and many benefits, but there needs to be improved communication and integration of local clinicians in a multidisciplinary manner for tele-medicine to realize its full potential. We can do this by implementing a shared health record amongst local clinicians and tele-neurologists so that both immediate and remote providers can have quick access to patient data and relevant health information suggested by the other providers [11]. There should also be active communication between local clinicians and the tele-neurologists, as well as active use of a central tele-health system. For instance, if a tele-health patient is seen by different specialists (i.e., neurology, rheumatology, ophthalmology), then each of these specialists should enter their notes into a central health record and be able to communicate with one another regarding the patient’s care.

This efficiency of care can be extended to where the patient is seen; if there is a remote facility for audio-visual conferencing, then this one patient should be able to be seen by different tele-health specialists at that one visit. Furthermore, perhaps local clinicians or internists can be involved at this facility as well; the patient can be examined by multiple specialists and their regular internist all in one visit. This will enable less traveling to the sites, as well as comprehensive, integrative, and multidisciplinary care that can be compiled with the assistance of their regular physician.

Although this would be key to a successful integrative network, tele-neurology must be examined in case-to-case bases. The type of tele-neurology provided, whether through audio-visual conferencing or through asynchronous forms such as email consultation, depends on the scenario. In a rural town, with the ability to support tele-neurology, then audio-visual conferencing could be incredibly useful if there is need and desire for more accessible neurology services amongst providers and patients. In this case, the integrative network of being seen by multiple tele-health providers can make an impactful difference in the care of people who would otherwise be burdened by long travel times and exorbitant costs.

However, in international, underdeveloped, or impoverished locations, this may not be entirely possible, and some other form of tele-neurology and tele-medicine may be more useful. In India, for instance, there are nearly one billion people who live in areas without a practising neurologist, while in Africa, the average population exceeds five million per one neurologist [7]. There is thus, a huge need in which tele-neurology has the potential to improve care and increase neurological services in these locations. However, Internet access in many of these locations, although growing, is sparse and two-way videoconferencing may not be possible or widely-available to implement. Asynchronous forms of tele-neurology, such as phone or email consultations, may be more possible. A prior review has proposed setting up a trans-continental, inter-regional, intra-regional, and a national network of neurologists utilizing tele-neurology to improve access to neurology care in Sub-Sarahan Africa. In this proposal, tele-education of local providers and tele-consultations between neurologists would take place with the hope that local neurologists can seek advice from colleagues within their own country, sub-region of Africa, or through neurologists in high-income countries [19].

This sort of model is most feasible since the local provider is most familiar with local site resources and disease epidemiology [6-7]; this model is also inexpensive and can take place with interrupted internet service or through a mobile phone. This model can also be extended so that further educational initiatives are implemented and neurologists who obtain training abroad or through tele-education can return to start other programs and provide further care in their home country. There could also be regular clinical case conferences to enforce collaborative relationships or urgent clinical consultations between these remote areas and global colleagues [7]. Below is a brief review on previous studies related to tele-neurology (Table 3).

**Table 3:** Brief review on previous studies related to tele-neurology CBOC: community-based outpatient clinic; ISDN: integrated services digital network; ED: emergency department; AIS: acute ischemic stroke; NIHSS: National Institutes of Health Stroke Scale; IV rt-PA: intravenous recombinant tissue plasminogen activator; ICH: intracerebral hemorrhage; ECASS: European Cooperative Acute Stroke Study; CI: confidence interval; TCT: telehealth clinical technician; CVT: clinical video telehealth; TIA: transient ischemic attack; DTN: door-to-needle; UAMC: University of Arkansas for Medical Sciences; NCC: neurocysticercosis; ICP: intracranial pressure; FIM: functional independence measure.

Study, Country	Clinical conditions (Health care providers)	Sample Size and Timeline of study	Methods and Setup	Outcomes and Results
Davis et al. 2012 [9], USA	Veterans with chronic neurologic conditions like Parkinson's disease, seizure disorders etc. (CBOC personnel and Neurologists)	354 patients from April 1, 2011 to March 31, 2013	Methods: The clinical video telehealth (CVT) system enables a neurologist to directly talk to and examine a Veteran with a neurologic illness at his or her local community-based outpatient clinic (CBOC). Setup: Tandberg or Jabber camera system at different participating sites and ISDN lines transmitted the information between the two sites at 548 kilobits/s.	Outcomes: Cost analysis and Patient Satisfaction. Results: 90% Patient satisfaction equivalent in both groups in the 5-level Likert scale questions. An average time of 5 hours and 325 miles and a total of $48,000 were saved.
Mazighi et al. 2015 [22], Paris	Acute Ischemic Stroke (AIS) (ED Physicians and Neurologists)	47 patients were randomized to usual care (n = 22) and tele-thrombolysis (n = 25) arms from April 2006 to March 2010.	Methods: After evaluation by the ED physician using the National Institutes of Health Stroke Scale (NIHSS) in the hospital without stroke unit facility, the stroke neurologist at the Bichat Stroke Center was contacted by a video-conferencing system to confirm the indication to use IV rt-PA for a suspected AIS patient. After confirmation patients were included in the study and randomly assigned to a usual care arm or a tele-thrombolysis arm. Setup: Video-conferencing.	Outcomes: Excellent outcome (modified Rankin scale (mRS) 0–1 at 90 days). Favorable outcome (90-day mRS 0–2) and early neurological improvement (NIHSS score 0–1 at 24 hours or a decrease of54 points within 24 hours). Safety outcomes included symptomatic intracerebral hemorrhage (ICH) per ECASS II definition, any ICH and all-cause mortality. Results: 15 patients (32%) reached an excellent outcome. Tele-thrombolysis arm group less frequently had an excellent outcome than usual care arm (16% vs. 50%, p = 0.013). After adjustment for age and prerandomization, NIHSS score this difference remained non-significant. Pre-randomization NIHSS was significantly associated with a less excellent outcome, with a multivariate OR per point increase of 0.75 (CI, 0.60–0.93; p<0.009). The multivariate ORs were 0.51 (CI, 0.12–2.17; p=0.36) for the tele-thrombolysis group, 0.96 (CI, 0.92–1.01; p=0.09) for age and 0.83 (CI, 0.71–0.96; p=0.014) for pre-randomization NIHSS. There were no significant differences between the two groups for early neurological improvement outcome and safety outcome.
Schreiber et al. 2018 [26], USA	Veterans with chronic neurologic conditions like Parkinson's disease, seizure disorders etc. (Neurologists and telehealth clinical technician (TCT))	745 encounter that included 570 unique patients between November 2011 and December 2014.	Methods: Teleneurology encounters were performed through clinical video telehealth (CVT), the teleneurologist assessed mental status, cranial nerves (motor functions), abnormal movements, coordination, and gait. The TCT assisted with evaluation of sensory function, muscle strength, tone, and tendon reflexes. In a random sampling of veterans, they were asked to complete a patient satisfaction survey. The survey was developed by the Teleneurology program and followed a Likert-like format. Setup: Community based outpatient clinic (COBC's) equipped with GlobalMed Mobile Telemedicine Station (GlobalMed, Scottsdale, AZ) with codec, camera, with pan/tilt/zoom capability remotely controlled by the provider, and two 21.5″ touchscreen monitors (resolution: 1,920 × 1,080). The provider site equipped with a Cisco TelePresence EX90 (Cisco Systems, San Jose, CA) with codec, camera, and 24-inch LCD monitor (resolution: 1,920 × 1,200, bandwidth capacity 386 kilobits per second).	Outcomes: To assess the feasibility (patient satisfaction and cost analysis) of applying telehealth modality to patients with chronic neurological disorders living in an urban setting. Results: The average age was 62.6 years and 3.5% were female. Greater than 90% of the respondents were satisfied with the care they received. 84% of the respondents preferred to have a CVT encounter at the CBOC rather than travel to the medical center. 86% would recommend a telehealth encounter with other veterans. 7% of respondents preferred a face-to-face encounter with a neurologist. There were an average cost savings of $6,276 during the study period.
Patterson et al. 2001 [28], UK and Bangladesh	Neurological disorders (Neurologists in UK and Local staff and doctors at Bangladesh)	Twelve consultations within 12 months	Methods: A small team of dedicated local staff were trained in the use of the equipment and how to send email referrals to a series of UK specialists. The use of a numbering system ensured patient confidentiality. Setup: Two digital cameras (CI400XL) and accessories, two tripods and a laptop.	Outcomes: Store and Forward Teleneurology method efficacy for delivering expert neurological advice. Results: Two cases were completed in one day, five in one week and 10 in three weeks. The neurologist would have preferred a video-link in eight of the 12 cases which he perceived as extremely complicated. The local doctors found the advice beneficial in 6 of these 8 cases as well as in the four more straightforward cases.
Cutting et al. 2013 [34], USA	Neurologic disorders (ED physicians and vascular fellowship trained neurologists)	498 patients were evaluated by Telestroke between March 2011 and March 2013.	Methods: Four urban hospitals (4 spokes) were included. Prior to Telestroke initiation, Emergency department (ED) protocols and rate of tPA administration were reviewed with an administrator and Neurologists practising at these spoke hospitals. When suspected AIS patients presented to a spoke, the ED physician called a central number, linked to the phone of the neurologist on-call. After video patient evaluation and review of imaging, recommendations regarding tPA were communicated to the ED and followed the American Heart Association/American Stroke Association guidelines. Setup: InTouch Health products and services.	Outcomes: Patient characteristics, time to initiation of the consult, and treatment decisions. Results: The mean age was 64.5 years, and 60.4% were female. Median time from initial emergency room call to start of Teleconsult was 5 (range, 1–51) minutes. The average length of Teleconsult was 30 minutes. 281 Telestroke patients (56.4%) were determined by Teleconsult to have an AIS or TIA. The tPA was recommended for 72 patients (14.5% overall; 25.6% of ischemic stroke/TIA patients). Transfer to the hub hospital occurred in 75 patients.
Kramer et al. 2014 [12], USA	Acute neurologic conditions (Resident Physicians, fellows and Neurologists)	36 trainees and 10 faculty members worked from July 2009 through November 2011. invited to participate via email	Methods: Resident physician and fellow trainees and faculty at a single institution who provided service over 29 months were surveyed. Responding participants answered 10 questions using a 5-point Likert scale or ranking. Setup: Surveys via email.	Outcomes: The survey compared experiences using the supervisory methods of telephone, robotic telepresence (RTP), and in-person interaction. Results: Surveys were received from 20 of the trainees (55.5%) and 8 of the faculty members (80%). 85% of both trainees and faculty preferred in-person supervision most, with robotic telepresence RTP ranked second and telephone being least favored. 38% of faculty and 70% of trainees reported telephone method was unsatisfactory for patient data review.
Belt et al. 2016 [35], USA	Epilepsy in Children	89 Stroke patients evaluated by in-transit Telestroke (ITTS) from January 2015 through March 2016.	Methods: ALS Units are easily clamped onto BLS ambulance stretchers and transmitted images during patient transport. Paramedics, trained for neurological emergencies assisted remote teleneurologists in obtaining a simplified history and examination, then coordinating care with the receiving emergency department. Setup: 4 ALS units were provided with an InTouch Xpress device, a portable unit incorporating a high-definition camera, microphone, and screen allowing transparent bidirectional communication.	Outcomes: Door-to-needle (DTN) and last-known-well-to-needle (LKWTN) times for all intravenous alteplase–treated stroke patients were assessed and compared in with and without in-transit Telestroke. Results: All alteplase-treated strokes brought to the ED had ITTS. Mean DTN time was 28 minutes (95% CI, 23–35) in ITTS patients, 41 in controls (95% CI, 36–47; P=0.02). Mean LKWTN time was 30 minutes less, 92 with ITTS (95% CI, 69–115), and 122 without (n=71; 95% CI, 109–135; P=0.037. Among non-ITTS patients evaluated by telemedicine (n=36), DTN was 12 minutes longer than with in-person assessment (n=48; P<0.001).
Bashiri et al. 2016 [36], USA	Neurological disorders (Neurologists)	Surveyed all patients at UAMC Neurology Clinic between March 2011 and December 2012.	Methods: The questionnaire was composed of the following four main questions- 1. Do you travel more than 1 hour by car to get to the neurology clinic? 2. Does your neurological condition make driving or travel difficult? 3. Have you missed appointments due to travel-related problems? 4. Does travel to the clinic create a financial hardship for you? Setup: Surveys	Outcomes: To assess patient interest in participating in Teleneurology for routine follow-up visits as well as demographic and medical factors associated with interest. Results: Of 1,441 respondents, 52.4% were interested in telemedicine. Of those interested versus uninterested in telemedicine, respectively, 68.9% versus 36.32% traveled more than 1 h to the clinic (p < 0.001), 64.7% versus 35.3% had difficulty secondary to neurological conditions (p < 0.001), 22.6% versus 6.8% had missed medical appointments due to travel problems (p < 0.001), and 43.1% versus 9.4% had travel-imposed financial hardship (p < 0.001).
Al Kasab et al. 2017 [13], USA	Acute Ischemic Stroke (Neurologists)	A total of 7,694 stroke consults at Medical University of South Carolina (MUSC) from May 1, 2008, through April 6, 2016	Methods: Retrospective Data was collected which included National Institutes of Health Stroke Scale (NIHSS) on presentation, number of IV alteplase administrations, number of patients transferred to MUSC, number of mechanical thrombectomies performed on transferred patients, the rate of symptomatic intracerebral hemorrhages (sICHs), and discharge location. Setup: Web conferencing.	Outcomes: To assess how the Telestroke program had developed and the rate and safety of (IV) alteplase administration through Telestroke. Results: Over the study period, the number of participating sites increased from 6 to 19 sites. The percentage of transfers decreased from 48% to 16%. Of the 7694 total, 3,795 (49.2%) patients were diagnosed with ischemic stroke, of those 1,324 (34.8%) received IV alteplase. The sICH occurred in 33 patients who received alteplase, and in 5 patients receiving a combination of IV and intra-arterial thrombolysis. The number of rtPA administered over Telestroke increased from 28 cases in 2008 to 336 cases in 2015. Average door-to-needle times decreased from 98.8 min in 2008 to 66.5 min in 2015.
Elson et al. 2018 [11], USA	Neurological disorders (Primary Care Physicians and Neurologists)	183 local clinicians surveyed, 89 completed the survey (43 among those in the usual care group and 46 in the intervention group) between March 2014 and August 2016.	Methods: Clinicians whose patients with Parkinson’s disease had participated in a national randomized controlled trial of video visits were Surveyed. Setup: Videoconferencing Polycom software on Dell notebook computers.	Outcomes: To determine whether clinicians received recommendations from remote specialists; recommendations were implemented; what barriers to speciality care local clinicians perceived; and recommend video visits. Results: Among respondents in the intervention group, 19 (44%) reported receiving the consultation note from the remote specialist. Sixteen (84%) of these implemented recommendations and 13 (81%) reported that the recommendations improved their patient’s condition. Thirty-six (40%) local clinicians would recommend video visits to their other patients with Parkinson’s disease. Thirty-two (37%) were unsure, and 12 (13%) would not recommend them. There was no difference in responses between those clinicians whose patients were in the usual care or the intervention group, nor between primary care physicians, neurologists, and Parkinson’s disease specialists.
Konanki et al. 2018 [15], India	Neurocysticercosis (NCC) and seizures ( Pediatrics Neurologist resident trainee)	402 children attending Neurocysticercosis (NCC) clinic between January –June 2011	Methods: The parents of children aged between 2–15 years with mild NCC burden (1–5 ring-enhancing lesions) and seizures, not receiving cysticidal drugs currently (Not eligible for cysticidal therapy or have completed cysticidal treatment), were contacted by a Pediatric Neurology Resident on Telephone before the scheduled hospital visit and were seen next day at the hospital by different Pediatric Resident. Setup: Structured Questionnaire.	Outcome: The primary objective was to estimate the diagnostic accuracy of telephone consultation to identify Critical Clinical Events compared with the Face-to-Face consultation (gold standard), in children with NCC and symptomatic seizures following the completion of cysticidal therapy. Results: Among 228 consultations, a total of 55 events were identified in 43 patients by Face-to-Face consultation (gold standard). Overall, 43 out of 228 consultations had Critical Events (18.8%). Among the individual groups of Critical Events, telephone consultation accurately identified seizures in all 18 children (sensitivity 100%), and raised ICP in all eight children (sensitivity 100%).
Dallolio et al. 2008 [37], Italy	Spinal Cord Injury (medical and nursing staff, physiotherapists, and occupational therapists)	137 study participants were recruited at 4 spinal cord units between November 2003 and February 2006. Randomized: 65 in telemedicine group and 68 in the control group	Methods: All patients received standard care from the spinal cord unit. In addition, patients randomized in the telemedicine group received 8 telemedicine weekly sessions in the first 2 months, followed by biweekly telemedicine sessions for 4 months. Telerehabilitation was performed by use of a dedicated videoconferencing platform. Setup: 1 central unit (set-top box), 1 webcam, 1 microphone with noise and echo cancellation, 1 remote controller, 1 universal serial bus electronic security key, 1 audio/video television connection cable and the related adapter, 1 power cable, 2 audio interconnection cables, 1 untwisted cable to connect to the integrated services digital network socket or to the asymmetric digital subscriber line modem, and 1 system reference manual.	Outcomes: To compare the Functional status at 6 months, clinical complications during the postdischarge period, and patient satisfaction of telerehabilitation intervention with those of standard care for spinal cord injury (SCI). Results: A higher improvement of functional scores in the telemedicine group was found only at the Italian site. FIM total score 3.38±4.43 (controls) versus 7.69±6.88 (telemedicine group), FIM motor score 3.24±4.38 (controls) versus 7.55±7.00 (telemedicine group) P .05 satisfaction with care was reported by patients in the telemedicine group across all sites.
Winter et al. 2019 [38], Germany	Stroke symptoms mimicked by actors (Paramedics and Neurologists)	40 Scenarios each group of 3G and 4G were used.	Methods: Trained actors presented stroke symptoms and paramedics assisted the remotely guided extended National Institutes of Health Stroke Scale (eNIHSS) assessment on the mobile stroke unit. Setup: Unit with camera, microphone and screen allowing use of bilateral communication.	Outcomes: To compare technical parameters of 4G and 3G connections, assessed the audio-visual quality of examination, and analyzed the reliability of neurological assessment and treatment decisions made by the remote neurologist versus the mobile stroke unit neurologist. Results: The remote examiners graded audio and video quality in 4G better than in 3G with slightly shorter assessment duration in 4G (mean: 9 (SD:5) vs. mean 11 (SD:3) min, p = 0.10). Reliability of the eNIHSS sum scores was high with intraclass correlation coefficients of 0.99 (95% CI: 0.987-1.00) for 4G and 0.98 (95% CI: 0.96-0.99) for 3G. None of the remote treatment decisions differed from onsite decisions.

## Conclusions

Tele-neurology is a powerful and innovative tool which enhances healthcare in the era of a global shortage of neurologists and other health care providers. Patients receive benefits of time-saving and reduced travel expenses. A physician also saves time, get exposed to a more diverse patient population, and has fewer missed appointments and cancellations. Utilization of tele-neurology has increased exponentially and is widely used for stroke, epilepsy, neurorehabilitation, pediatric neurology, outpatient consultation, drug refills, mental health evaluation, and global neurology. Though some experts believe that tele-neurology has its limitations, overall it is an effective tool to bring a new horizon in modern neurological care. This review focuses on the the trajectory of tele-neurology utilization and the issues that need to be addressed so that underserved communities can benefit from it in the near future.
